# Disease specific symptoms indices in patients with celiac disease—A hardly recognised entity

**DOI:** 10.3389/fnut.2022.944449

**Published:** 2022-09-07

**Authors:** Shaista Jabeen, Azmat Ullah Khan, Waqas Ahmed, Mansur-ud-Din Ahmad, Saghir Ahmad Jafri, Umar Bacha, Amjed Ali, Hafiz Shehzad Muzammil, Suliman A. Alsagaby, Waleed Al Abdulmonem, Mohamed A. Abdelgawad, Mishal Riaz, Mahwish, Makia Nasir, Aimen Zafar, Tabussam Tufail, Muhammad Imran, Tallat Anwar Faridi, Maria Aslam, Syedda Fatima Abid Shah, Sana Farooq, Tayyaba Niaz Awan, Habib Ur-Rehman

**Affiliations:** ^1^Department of Food Science and Human Nutrition, University of Veterinary and Animal Sciences, Lahore, Pakistan; ^2^University Institute of Diet and Nutritional Sciences, Faculty of Allied Health Sciences, The University of Lahore, Lahore, Pakistan; ^3^Department of Epidemiology and Public Health, University of Veterinary and Animal Sciences, Lahore, Pakistan; ^4^Nur International University, Lahore, Pakistan; ^5^Department of Nutrition Sciences, University of Management and Technology, Lahore, Pakistan; ^6^Faculty of Allied Health Sciences, University Institute of Physical Therapy, The University of Lahore, Lahore, Pakistan; ^7^National Institute of Food Science and Technology, University of Agriculture Faisalabad, Faisalabad, Pakistan; ^8^Department of Medical Laboratory Sciences, College of Applied Medical Sciences, Majmaah University, Al Majmaah, Saudi Arabia; ^9^Department of Pathology, College of Medicine, Qassim University, Buraydah, Saudi Arabia; ^10^Department of Pharmaceutical Chemistry, College of Pharmacy, Jouf University, Sakaka, Saudi Arabia; ^11^Institute of Home Sciences, University of Agriculture Faisalabad, Faisalabad, Pakistan; ^12^The Physio College of Rehabilitation, Multan, Pakistan; ^13^University Institute of Food Science and Technology, The University of Lahore, Lahore, Pakistan; ^14^Department of Food Science and Technology, University of Narowal, Narowal, Pakistan; ^15^Food, Nutrition and Lifestyle Unit, King Fahed Medical Research Center, Clinical Biochemistry Department, Faculty of Medicine, King Abdulaziz University, Jeddah, Saudi Arabia; ^16^Faculty of Allied Health Sciences, University Institute of Public Health, The University of Lahore, Lahore, Pakistan; ^17^Health Services Academy, Islamabad, Pakistan; ^18^Hussain Memorial Hospital, Lahore, Pakistan

**Keywords:** celiac disease, anemia, wasting, hypoalbuminemia, gastrointestinal discomforts

## Abstract

**Background:**

Celiac disease (CD) was considered a rare disease before and was perceivably only limited to children but now affects almost 1–2% of the global population. This abrupt increase in prevalence is due to advancements in diagnostic criteria and medical facilities but still many countries lack the basic data that can assess the severity of this health issue. The present study was conducted with the aim to assess the common but rarely diagnosed condition with the identification of its underlying secondary ailments.

**Materials and methods:**

Patients visiting public sector hospitals were recruited and tested for clinical symptoms secondary to gluten-containing foods (wheat and barley, etc.), followed by serological testing for immunoglobulin A, tissue transglutaminase A, and anti-endomysial antibodies. Only seropositive candidates were included in the endoscopic and biopsy examination for the features of villous atrophy and intestinal cell damage. The secondary ailments including anemia, growth retardation, and gastrointestinal symptoms were also documented for the tested positive patients. The modified European Society of Pediatric Gastroenterology, Hepatology, and Nutrition (ESPGHAN) criterion was followed throughout the study.

**Results:**

From 647 suspected cases from March 2018 to July 2019, 113 were confirmed with CD while 58% were female children and 42% were male children. The majority of them were from a lower class (75%) and 26% of them had a positive family history of CD. A total of 67% of patients with CD were underweight while wasting was observed in 38%, and 80% were stunted as well. Of the positively tested patients with CD, 49% had moderate anemia with 15% having severe anemia. Approximately 33% had hypoalbuminemia as well. The majority of them had a mild to severe range of gastrointestinal symptoms, such as abdominal pain, diarrhea, flatus, eructation, diarrhea, and steatorrhea.

**Conclusion:**

The study finding indicates an increased number of patients diagnosed with CD with an excessive sum of secondary ailments, such as anemia, growth failure, growth retardation, malnutrition, and gastrointestinal symptoms.

## Introduction

Celiac disease (CD) is an autoimmune systemic enteropathy that can be triggered by the ingestion of gluten proteins (mainly from wheat, rye, and barley) with manifestation in the small intestine and extra interstitial organs prevalent in almost all age groups (1, 2). Earlier CD was considered a rare disease that only occurred in children. According to John Walker-Smith and Gee, CD was some kind of chronic indigestion affecting children between the ages of 1–5 with symptoms of foul-smelling feces, pale in color, and bulk in nature. These concepts were altered by the findings of Dicke, reporting gluten as the causative agent for CD, and it can be caused by all cereals, especially wheat flour. Later on, mucosal lesions, i.e., villous atrophy and crypt hyperplasia were identified as diagnostic features in CD (3). The onset of the disease was perceived to be gradual with loss of muscle and fat (cachexia), failure to thrive, and mal-absorption syndrome (4). The advances in diagnostic criteria and true understanding of the nature of disease made the scientists realize and calculate the true prevalence of CD (5). For more than 2 decades, CD has emerged as a major public health concern with an initial prevalence of 1% reported by various European countries (6).

Patients with CD may also present with adjacent underlying complications, i.e., failure to thrive, short stature, delayed puberty, tiredness, loss of weight, muscle mass, and fat mass but 10% of the patients with CD can be obese, and therefore they should not be overlooked. CD may also present with various gastrointestinal complaints, i.e., diarrhea, cramping, bloating, flatulence, nausea, and electrolyte imbalance (7, 8). There are certain non-classical symptoms of CD, such as iron deficiency anemia, increased transaminases, constipation, ataxia, lethargy, osteoporosis, and dyspepsia (9). Currently, the diagnostic criterion of CD is based on the guidelines described in the European Society of Pediatric Gastroenterology, Hepatology, and Nutrition (ESPGHAN). The criterion includes documentation of history, serology, and histology. Marsh measures are used in the ESPGHAN for histology interpretation and CD confirmation including attenuation of duodenal folds with crypt hyperplasia and excessive aggregation of intraepithelial lymphocytes (10).

The prevalence of CD has been increasing with the passage of time (0.6% from 1991 to 2000 and 0.8% from 2000 to 2016) but still missing data from developing countries, such as Pakistan. A systemic review and meta-analysis conducted to assess the global prevalence of CD suggested various readings including a 1.4% seroprevalence of CD based on 275,818 individuals (95% *CI*; confidence interval, 1.1–1.7%) but Asia was among the highest CD prevalent region (1.8%) from the whole. The seroprevalence was based on the quantification of anti-tissue transglutaminase and anti-endomysial antibodies. Based on 138,792 individuals, it was reduced up to 0.7% (95% *CI*, 0.5–0.9%) based on the biopsy confirmation having clear indications of villous atrophy and crypts hyperplasia. European countries were found to have the highest CD prevalence with 0.8% along with Asia (0.6%). CD prevalence was 0.5% in North America and Africa. The least prevalence of 0.4% was found in South America. The overall proportion of CD was more among female children compared with male children (female children 0.6%, male children 0.4%, *p* < 0.01) with a ratio of 1:3 (male:female) (11) and children were the most affected age group compared with adults (children 0.9%, adults 0.5%, *p* < 0.01) (12). A province (Asia) based study reported 1.6% of seroprevalence of CD and 0.5% based on biopsy parameters (13). The Human Leukocyte Antigen on alleles DQ2 and DQ8 (heterodimeric; surface receptors) have great significance in the CD diagnosis. The prevalence also varies based on these high-risk populations (1.2–55% CD) and low-risk populations (0.14–5.7%).

Due to the multisystem nature of the disease with a lot of expensive and invasive diagnostic procedures, the majority of cases go undiagnosed, and the chances of having a false positive also increase because of the high level of doubt (9). Among the top 10 most populated countries around the globe, only 4 of them (US, India, Brazil, and Russia) had population-based data on the prevalence of CD. Pakistan, China, Indonesia, Nigeria, Bangladesh, and Japan lack the data, although CD has been reported in all these countries except Nigeria (12). Various studies conducted to assess the prevalence of CD along with existing medical conditions, such as anemia and short stature, are available in the literature. A longitudinal study conducted to assess the CD among the children with the study duration of years reported a 60.03% prevalence. Approximately 26% of them were anemic, 90.7% were underweight, 83.9% had short stature, and 40–50% reported diarrhea, abdominal pain, and distension (14). A cross-sectional study conducted in the public sector hospital in Lahore reported a 12.1% prevalence of CD with 80.3% having iron deficiency anemia (IDA) (15) in another study prevalence was 28.2% with 53.8% having macrocytic anemia and 38.46% having microcytic anemia (16, 17).

All these studies either had a low sample size or were carried out with reference to an existing underlying clinical manifestation. In some of the studies, only seroprevalence was measured and ESPGHAN criteria were not followed. The prevalence of all typical and atypical symptoms was not considered in these studies also. Therefore, the present study was conducted to assess the prevailing gastrointestinal symptoms, signs of growth failure, anemia, and cachexia in patients presenting in a public and private sector hospital in Lahore city-Pakistan.

## Materials and methods

The present study was a descriptive cross-sectional survey conducted to identify the patients with CD or person along with prevailing gastrointestinal complaints, signs of growth failure, and malnutrition.

### Consent and ethical considerations

The study protocol was approved by the Ethical board for biomedical research, the University of Veterinary and Animal Sciences, Lahore, Pakistan. (No.029/IRC/BMR) Written informed consent was obtained from the parents of children or the legal guardian before enrolling any participant in the study.

### Study participants

**Figure 1:**
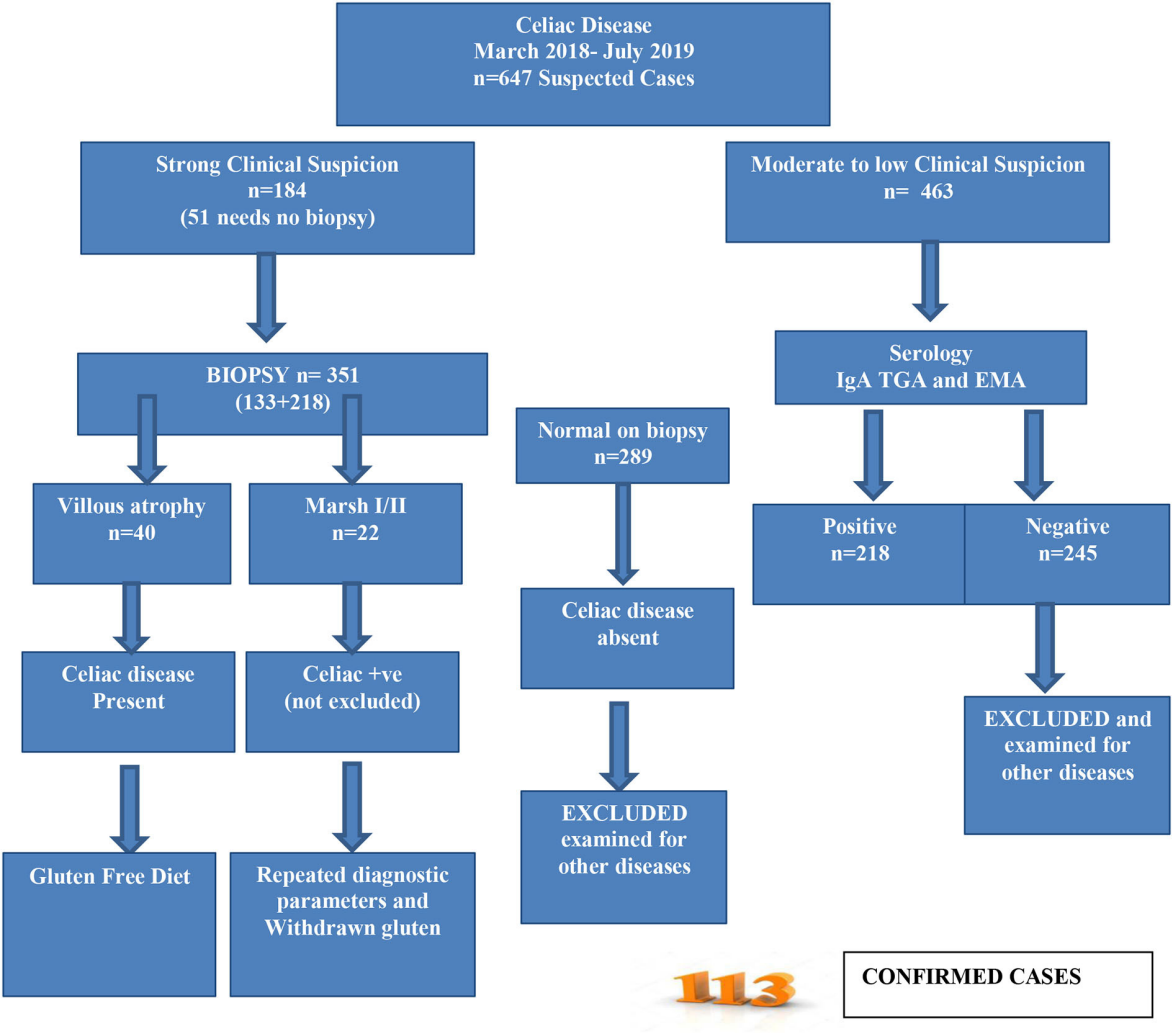
Diagnosis and screening of celiac patients according to ESPGHAN criteria.

Patients with celiac disease/gluten sensitivity were recruited according to the modified ESPGHAN criteria (Figure 1) (18). A total of 647 participants suspected of CD were enrolled for further assessment. A gluten-free diet was recommended for the confirmed CD cases immediately.

### Nutritional and clinical assessment

Screening of the patients was done through symptoms ranging from classic signs of a mal-absorption syndrome, such as diarrhea (increase in liquidity and frequency than normal for >2 weeks), weight loss (weight for age below 5th percentile), growth failure (linear height below the 5th percentile for age), and anemia (pallor) to non-specific symptoms, such as chronic constipation or abdominal pain.

### Anthropometry

Body weight was measured using a weighing scale with standardization done after every 20 readings. Height was measured (without shoes) in the position, back and hips touching the wall by using wall-mounted stedio-meter. The mid-upper arm circumference (MUAC) was measured by MUAC measuring tape from the midpoint between the acromion process of the scapula and the tip of the elbow. The body mass index (BMI) was calculated as weight in kilograms divided by height in meter square (19). The WHO standard growth charts, designed separately for boys and girls were used as a research tool to assess their nutritional status. A patient adherent to a gluten-free diet and its duration without positive serologic test results for CD or diagnosed by the physician were identified by age and sex also.

### Serology

Celiac disease was defined as having either double-positive serologic test results on IgA, tissue trans-glutaminase through ELISA, or a reported diagnosis by a healthcare professional coupled with gluten-free diet consumption. On the basis of symptoms, in the case of suspected CD, serum levels of IgA autoantibodies to tissue trans-glutaminase IgA (tTG-IgA) were determined (20).

### Endoscopy

Those individuals who were found to be positive for ELISA were contacted for further tests as per protocol. A detailed clinical and hematological evaluation, complete blood count (CBC), and biochemical tests of liver enzymes were conducted. Upper gastrointestinal and duodenal endoscopic examinations were done to check villous atrophy by using a video endoscope (21).

### Statistical analysis

Data obtained from the study were analyzed using a statistical package for social sciences (SPSS) and expressed as mean ± standard deviation (SD), frequencies, and percentages. A descriptive analysis was performed to obtain the before-mentioned data.

## Results

The present study was conducted to find the symptoms of gluten insensitivity/CD in both genders. Data related to demographics, anthropometry, diagnostic tests related to blood, and endoscopy were performed alongside the frequency of gastrointestinal complaints.

From the collected data, 58% were female children and 42% were male children. The majority of the participants were from the age group of 2–5 years and 6–9 years and only 2% were from the age group of >15 years with a mean age of 7.27 years. Almost three-fourths of the study participants had their own houses and 27% were living in rental houses. The majority of the study participant's fathers were either laborers (39%) or had private jobs (34%), and 11% were government servants. Almost half of the participant's family income was between 20,000 and 50,000 PKR and only 17% had a family income of more than 50,000 PKR in the collected data. All the participants either belonged to the lower or middle socioeconomic class with 75% from the lower socioeconomic class (Table 1).

**Table 1:** Demographic profile of patients with celiac disease (CD).

**Gender**	**Frequency (*n*)**	**Percent (%)**
Male	47	41.6
Female	66	58.4
**Age (Years)**		
2-5	49	43.3
6-9	36	31.8
10-15	26	23
>15	2	1.8
**Age (Years)**; Mean ± SD	7.27 ± 3.68	
**Residence**		
Own house	83	73.4
Rental	30	26.6
**Father's occupation**		
Laborer	44	39
Farmer	17	15
Govt. job	13	11.5
Private job	39	34.5
**Family's income/month (PKR:USD)**		
<20,000 PKR/ <100USD	46	40.8
20,000–50,000PKR/100–250 USD	48	42.4
>50,000PKR/>250–USD	19	16.8
**Socioeconomic Status**		
Lower class	85	75.2
Middle class	28	24.8

The majority of the study participants were undernourished, as their weight for age (percentile) was from <1st and 3rd percentiles, indicating under-nutrition, from which 38% were severely undernourished (<1st percentile) and 29% were from 3rd percentile. The same trend was observed in the height for age parameter, with 70% of the participants having <5th percentile in height for age and 8% were severely stunted (<1st percentile). Approximately 79% of the participants were of short stature (Table 2).

**Table 2:** Anthropometric measurements of patients with celiac disease.

Weight (Kg); **Mean ±SD**	17.29 ± 9.18	
Height (cm); **Mean ±SD**	103.5 ± 23.04	
Body mass index (BMI); **Mean ±SD**	15.43 ± 4.35	
**Weight for age (Percentile)**	**Frequency (** ***n*** **)**	**Percent (%)**
<1st	43	38.1
3rd	33	29.2
>5th	24	21.2
>15th	12	10.6
>75th	1	0.9
**Height for age (Percentile)**		
<1st	9	8
3rd	80	70.8
>5th	13	11.5
>15th	11	9.7
**Short stature**		
Yes	89	78.7
No	24	21.3

Of the total study population, 95% had positive levels of IgG and all of them had positive IgA levels for CD. Almost 15% of the participants had severe anemia and 50% had moderate level anemia while 70% of the participants had lower hematocrit levels. Approximately, 33% of the participants had hypoalbuminemia. Liver enzymes were increased in almost 34–36% of the participants (Table 3).

**Table 3:** Blood chemistry of patients with celiac disease.

**Parameters**	**Frequency (*n*)**	**Percent (%)**	**Mean ±S.D**
**Immunoglobulin A (tTG-IgA)**			142 ± 46
Positive	113	100	
**Hemoglobin**			9.5 ± 1.4
Normal >11 mg/dL	40	35.4	
Moderate anemia 8–10 mg/dL	56	49.5	
Severe anemia <8 mg/dL	17	15.1	
**Hematocrit (HCT)**			-
Normal >32–37%	34	30.1	
Low <32–37%	79	69.9	
**Albumin**			2.94 ± 0.99
Normal >3.3–3.8 g/dL	76	67.2	
Low	37	32.8	
**Serum glutamic pyruvic transaminase (SGPT)**			28.3 ± 6.4
Normal	69	61.1	
Low	4	3.5	
High	40	35.4	
**Serum glutamic-oxaloacetic transaminase (SGOT)**			35.2 ± 4.9
Normal	72	63.7
Low	2	1.8
High	39	34.5

Almost 46% of the participants had moderate abdominal pain, and 22% had severe abdominal pain. About 36% were having moderate heartburn and 5.5% had severe heartburn. Almost 33% had moderate regurgitation and 4% had severe regurgitation. About 38% were facing moderate nausea and 46% had no nausea. Approximately, 24% of the participants had moderate borborygmus and 63% of them had severe episodes. From the collected data, 67 and 53% of the participants had no Eructation and Flatus, respectively. Hard stool, urgency, and incomplete evacuation were absent in approximately 93–99% of the participants. Approximately 12% had severe episodes of steatorrhea (Table 4).

**Table 4:** Gastrointestinal complaints of patients with celiac disease.

**Gastrointestinal** **Complaints**		**Mild**	**Moderate**	**Severe**	**Absent**
Abdominal pain	Frequency (*n*)	18	50	24	17
	Percent (%)	16.5	45.9	22	15.6
Heartburn	Frequency (*n*)	29	39	6	35
	Percent (%)	26.6	35.8	5.5	32.1
Regurgitation	Frequency (*n*)	26	36	4	43
	Percent (%)	23.9	33	3.7	39.4
Nausea	Frequency (*n*)	8	41	10	49
	Percent (%)	7.3	37.6	9.2	45.9
Borborygmus	Frequency (*n*)	12	26	2	69
	Percent (%)	11	23.9	1.8	63.3
Eructation	Frequency (*n*)	9	24	3	73
	Percent (%)	8.3	22	2.8	67
Flatus	Frequency (*n*)	10	27	14	58
	Percent (%)	9.2	24.8	12.8	53.2
Diarrhea	Frequency (*n*)	7	49	25	28
	Percent (%)	6.4	45	22.9	25.7
Loose Stool	Frequency (*n*)	6	52	22	29
	Percent (%)	5.5	47.7	20.2	26.6
Hard Stool	Frequency (*n*)	1	0	0	108
	Percent (%)	0.9	0	0	99.1
Urgency	Frequency (*n*)	2	5	0	102
	Percent (%)	1.8	4.6	0	93.6
Incomplete Evacuation	Frequency (*n*)	3	6	2	98
	Percent (%)	2.8	5.5	1.8	89.9
Steatorrhea	Frequency (*n*)	7	39	13	50
	Percent (%)	6.4	35.8	11.9	45.9
Fever	Frequency (*n*)	0	73	36	0
	Percent (%)	0	67	33	0
Cough	Frequency (*n*)	0	34	75	0
	Percent (%)	0	31.2	68.8	0

**Figure 2:**
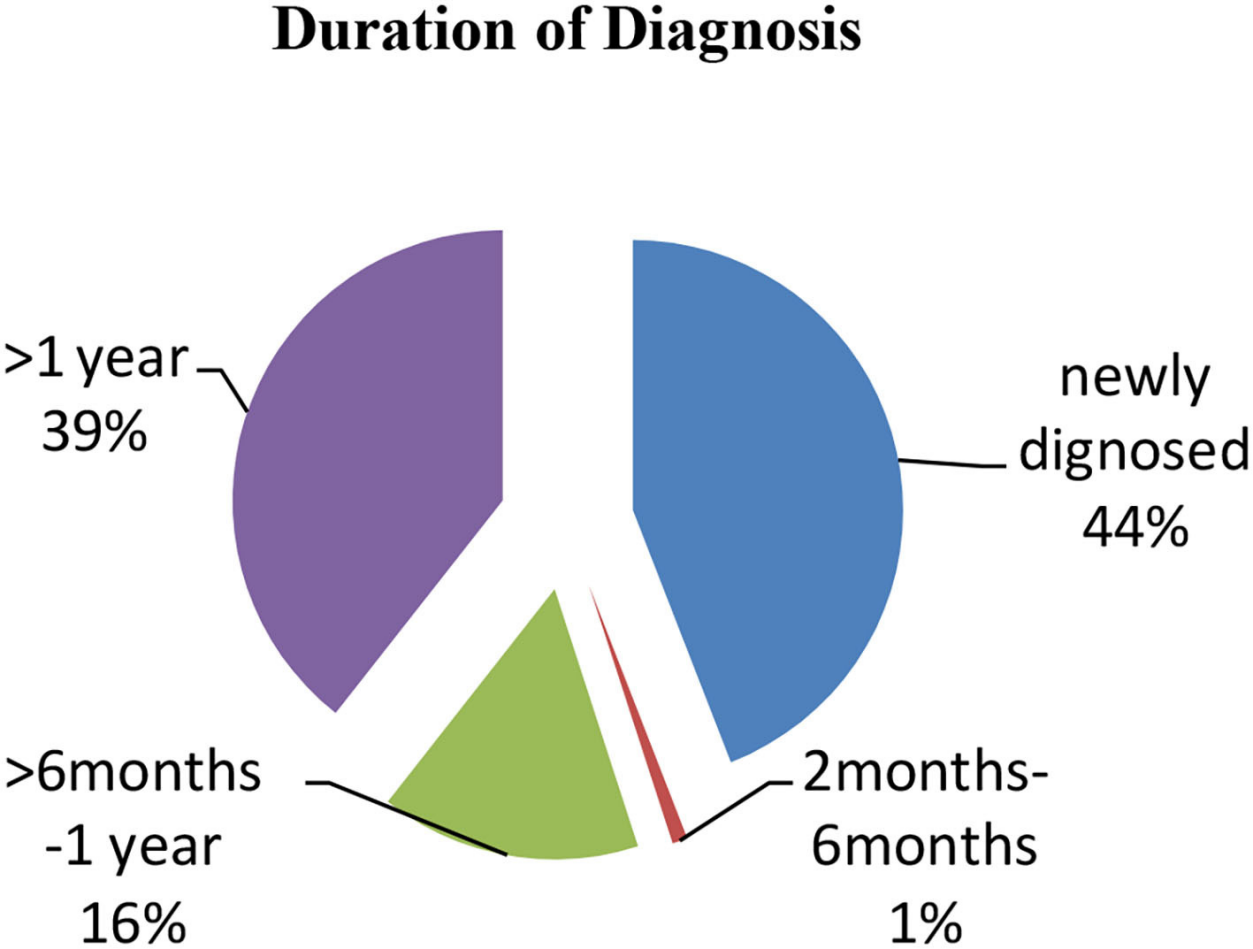
Distribution of patients with celiac disease (CD) according to the duration of diagnosis.

The majority of the participants were diagnosed with CD for more than 1 year presenting with clinical symptoms (39.4%) and approximately 16% were diagnosed for 1 month while 44% were newly diagnosed patients (Figure 2). Only 26% had a positive family history of CD. From the collected data, 20% of the participants showed Marsh I/II and 35% had Marsh III on endoscopic findings, which are clear indications of CD (Table 5). A total of 45% of participants did not undergo endoscopic examination due to strong clinical suspicion and responsiveness to a gluten-free diet.

**Table 5:** Disease history and endoscopic findings of patients with celiac disease.

**Family History**	**Frequency (*n*)**	**Percent (%)**
Positive	29	25.7
Negative	84	74.3
**Endoscopic findings**		
Not done	51	45.1
Marsh 1	20	17.7
Marsh 2	2	1.8
Marsh 3	40	35.4

## Discussion

The present study was conducted with the objective to assess the prevailing symptoms of CD with a special focus on the underlying conditions, such as anemia, malnutrition, and gastrointestinal symptoms. CD, also known as heterogeneous autoimmune disease, Is triggered when gluten is ingested. It was considered rare in early times, but now it is one of the common public health concerns. The global prevalence of gluten allergy is increasing rapidly now. Studies have shown the prevalence is higher in infants and children. Some studies highlighted that it may be due to the early introduction of gluten-related products (22) but some denied this fact. One Norwegian study concludes that gluten must be introduced to infants over 6 months of age (23). In Italy, one study is done with school-going children having CD and the results showed that prevalence in Italy is increased by 1.5% in the last 25 years (24). The present study showed that majorly children, 113 out of 647, affected due to this autoimmune disease belonged to low-income status. Of all of the participants, 113 had 10 times greater tTG-IgA levels (> 7 U/ml). Male children were 41.6% and female children were 58.4% with an M:F ratio of 1:1.4. Similar trend was observed in the study in which out of 350, 126 patients fulfilled the criteria including 54 male children and 71 female children with an M:F ratio of 1:1.3 (25).

This disease is responsible for a broad spectrum of symptoms and problems. Starting from malnutrition to failure to thrive, CD children are prone to many other underlying issues, such as gastrointestinal problems, anemia, liver problems, and many others (7). Most studies done in this context are proof of malnutrition in relation to CD. Mal-absorption contributes the most important role in causing malnutrition and growth problems in patients with CD (26). As a result of malabsorption, a lesser amount of substrate is available due to which the “energy compensation” mechanism in the body is activated. In this mechanism, the body stores fats (in adipose tissues), and proteins (in muscles) start to deplete. This whole process results in severe weight loss and retardation in the growth process. If it continues for 3–4 months, the weight loss will result in stunting (27, 28). All the infants and children are considered short-statured when compared with growth charts (24). Delayed puberty in CD adolescent girls is also of primary concern. Delayed bone aging and amenorrhea accompanied by infertility can be caused if the patient is not treated well-according to the condition (29). In our study, this fact was proved as more than 79% of participants were short stature and 88% were severely wasted as <5th percentile when growth charts were plotted. The mean BMI of the study participants was 15.43 ± 4.35.

Some common manifestations of the gastro-intestinal tract in celiac patients are abdominal distension accompanied by diarrhea, abdominal pain, anorexia, vomiting, and in some cases ulcers and binge eating (30). Overweight or children with excessive appetite get usually masked due to the presence of some other diseases with CD. The current study reported severe abdominal pain in 22% of cases while nausea was present in 9.2 and 70% of patients with CD were suffering from different degrees of diarrhea (mild, moderate, and severe). When reviewed in the literature, it is noted that abdominal distension is one of the most common symptoms faced by patients with CD (31, 32). Similarly, in (33) study, diarrhea is observed in more than 70% of patients with CD. Comparing facts with our present study, it was clear that such symptoms were also present in our targeted population in clear significant percentages.

The blood chemistry of patients with CD is also altered in a visible manner. Most of the patients with CD (65%) suffer from anemia as the main issue as well. Along with anemia, some other problems, such as major changes in the endocrine system of the patients are also seen (28). A significant inverse relationship between the exposure to gluten and insulin-like growth hormone 1 (IGF-1) has been studied. It is noticed that the secretion of this particular hormone is only affected when the gluten is exposed for a very long time. These changes lead to a decrease in the growth velocity. When the condition is worsened, it is studied that the growth hormone released from the hypothalamus is also affected (28, 34, 35). Due to malabsorption, anemia is very prevalent in patients with CD. Our study showed clear results of anemia in patients with CD. Some were highly anemic and a major population was moderately anemic. Along with lower hemoglobin levels, hematocrit levels were also lesser in patients with CD when the values were compared with normal people. With low hemoglobin levels, albumin levels are also affected in patients with CD. Due to malabsorption, despite of high protein diet, the patients do not respond to a protein diet hence leading to hypo-albuminemia in patients who are not treated properly. In addition, our study showed hypo-albuminemia in patients with CD.

For screening, some very reliable and sensitive tests are performed. These tests are specific screening tests; IgA anti-tissue Transglutaminase (IgA tTG antibodies) are prescribed for checking and confirming CD (36). These antibodies are generated when gluten is exposed to the small intestine and the auto-immune system gets triggered. The mucosal lining of the intestine is damaged due to immune-mediated response as the result of gluten intolerance (37). Our study showed that all the targeted patients were positive for this test showing the occurrence of CD. IgG immunoglobulin tests were also done and the majority of them showed positive results for these immune globulins. The liver is also affected by CD. Many researchers have found a relationship between CD and autoimmune liver injuries (38). More than 30% of participants in our study showed increased liver enzymes level. For diagnosis, endoscopic findings are considered. About 18–36% of the study participants had moderate to severe villous atrophy upon endoscopic and biopsy examination. The endoscopic examination is considered the gold standard invasive method for the diagnosis of CD (39). Compared with our findings, all 75 of the patients found positive with serological screening had prominent histological and endoscopic changes in the intestines in terms of villous atrophy (40).

## Conclusion

Population-based data on CD are missing for various underdeveloped/developing countries including Pakistan. It is not uncommon in our population. The present study found that a large number of celiac children aged between 2 and 9 years presented with anemia, diarrhea, growth failure, mal-digestion, and malabsorption. A total of 28% cases were reported with non-diarrheal CD. Disturbed serological titers and biopsy findings are hallmarks of the disease.

## Data availability statement

The raw data supporting the conclusions of this article will be made available by the authors, without undue reservation.

## Ethics statement

The studies involving human participants were reviewed and approved by Institutional Review Committee For Biomedical Research Uvas, Lahore, Pakistan. Written informed consent to participate in this study was provided by the participants' legal guardian/next of kin.

## Author contributions

All authors listed have made a substantial, direct, and intellectual contribution to the work and approved it for publication.

## Conflict of interest

The authors declare that the research was conducted in the absence of any commercial or financial relationships that could be construed as a potential conflict of interest.

## Publisher's note

All claims expressed in this article are solely those of the authors and do not necessarily represent those of their affiliated organizations, or those of the publisher, the editors and the reviewers. Any product that may be evaluated in this article, or claim that may be made by its manufacturer, is not guaranteed or endorsed by the publisher.
