# Corrigendum: Disease specific symptoms indices in patients with celiac disease—A hardly recognised entity

**DOI:** 10.3389/fnut.2022.1073102

**Published:** 2022-11-16

**Authors:** Shaista Jabeen, Azmat Ullah Khan, Waqas Ahmed, Mansur-ud-Din Ahmad, Saghir Ahmad Jafri, Umar Bacha, Amjed Ali, Hafiz Shehzad Muzammil, Suliman A. Alsagaby, Waleed Al Abdulmonem, Mohamed A. Abdelgawad, Mishal Riaz, Mahwish, Makia Nasir, Aimen Zafar, Tabussam Tufail, Muhammad Imran, Tallat Anwar Faridi, Maria Aslam, Syedda Fatima Abid Shah, Sana Farooq, Tayyaba Niaz Awan, Habib Ur-Rehman

**Affiliations:** ^1^Department of Food Science and Human Nutrition, University of Veterinary and Animal Sciences, Lahore, Pakistan; ^2^University Institute of Diet and Nutritional Sciences, Faculty of Allied Health Sciences, The University of Lahore, Lahore, Pakistan; ^3^Department of Epidemiology and Public Health, University of Veterinary and Animal Sciences, Lahore, Pakistan; ^4^Nur International University, Lahore, Pakistan; ^5^Department of Nutrition Sciences, University of Management and Technology, Lahore, Pakistan; ^6^Faculty of Allied Health Sciences, University Institute of Physical Therapy, The University of Lahore, Lahore, Pakistan; ^7^National Institute of Food Science and Technology, University of Agriculture Faisalabad, Faisalabad, Pakistan; ^8^Department of Medical Laboratory Sciences, College of Applied Medical Sciences, Majmaah University, Al Majmaah, Saudi Arabia; ^9^Department of Pathology, College of Medicine, Qassim University, Buraydah, Saudi Arabia; ^10^Department of Pharmaceutical Chemistry, College of Pharmacy, Jouf University, Sakaka, Saudi Arabia; ^11^Institute of Home Sciences, University of Agriculture Faisalabad, Faisalabad, Pakistan; ^12^The Physio College of Rehabilitation, Multan, Pakistan; ^13^University Institute of Food Science and Technology, The University of Lahore, Lahore, Pakistan; ^14^Department of Food Science and Technology, University of Narowal, Narowal, Pakistan; ^15^Food, Nutrition and Lifestyle Unit, King Fahed Medical Research Center, Clinical Biochemistry Department, Faculty of Medicine, King Abdulaziz University, Jeddah, Saudi Arabia; ^16^Faculty of Allied Health Sciences, University Institute of Public Health, The University of Lahore, Lahore, Pakistan; ^17^Health Services Academy, Islamabad, Pakistan; ^18^Hussain Memorial Hospital, Lahore, Pakistan

**Keywords:** celiac disease, anemia, wasting, hypoalbuminemia, gastrointestinal discomforts

In the published article, there was an error in affiliation 2. Instead of “Faculty of Allied Health Sciences, University Institute of Diet and Nutritional Sciences, The University of Lahore, Lahore, Pakistan,” it should be “University Institute of Diet and Nutritional Sciences, Faculty of Allied Health Sciences, The University of Lahore, Lahore, Pakistan.”

The authors apologize for this error and state that this does not change the scientific conclusions of the article in any way. The original article has been updated.

## Publisher's note

All claims expressed in this article are solely those of the authors and do not necessarily represent those of their affiliated organizations, or those of the publisher, the editors and the reviewers. Any product that may be evaluated in this article, or claim that may be made by its manufacturer, is not guaranteed or endorsed by the publisher.

